# Machine Learning Approach to Quadratic Programming-Based Microwave Imaging for Breast Cancer Detection

**DOI:** 10.3390/s22114122

**Published:** 2022-05-29

**Authors:** Sandra Costanzo, Alexandra Flores, Giovanni Buonanno

**Affiliations:** 1 Dipartimento di Ingegneria Informatica, Modellistica, Elettronica e Sistemistica, Università della Calabria, 87036 Rende, Italy; alexandra.flores@unical.it (A.F.); giovanni.buonanno@unical.it (G.B.); 2 Inter-University National Research Center on Interactions between Electromagnetic Fields and Biosystems (ICEmB), 16145 Genoa, Italy; 3 National Research Council of Italy (CNR), Institute for Electromagnetic Sensing of the Environment (IREA), 80124 Naples, Italy; 4 Consorzio Nazionale Interuniversitario per le Telecomunicazioni (CNIT), 43124 Parma, Italy

**Keywords:** inverse scattering, breast phantoms, convolution neural network, permittivity, strong dielectric scatterers, Born iterative method

## Abstract

In this work, a novel technique is proposed that combines the Born iterative method, based on a quadratic programming approach, with convolutional neural networks to solve the ill-framed inverse problem coming from microwave imaging formulation in breast cancer detection. The aim is to accurately recover the permittivity of breast phantoms, these typically being strong dielectric scatterers, from the measured scattering data. Several tests were carried out, using a circular imaging configuration and breast models, to evaluate the performance of the proposed scheme, showing that the application of convolutional neural networks allows clinicians to considerably reduce the reconstruction time with an accuracy that exceeds 90% in all the performed validations.

## 1. Introduction

Breast cancer is the most commonly reported disease in women around the world, surpassing lung cancer. Its incidence and related mortality rate have been increasing in recent years, so that, according to the GLOBOCAN worldwide statistics for 2020, it has been estimated that nearly 2.3 million women were then diagnosed with breast cancer [1]. Periodic controls for an early diagnosis are, thus, always a priority. Mammography is the most widely used method for diagnosing breast cancer, using X-ray ionizing radiation to generate images of breast tissue. However, it is not always recommended because exposure to X-rays can cause damage to cells; in addition, it has limitations when identifying those lesions with regions of dense glandular tissue. Several alternatives have been developed, such as ultrasound imaging, breast magnetic resonance imaging, and the automated breast volume scanner (ABVS) [2], but these methods have their own drawbacks, such as the presence of several false-positive results.

The use of microwaves to obtain images of the breast is another alternative to the previous methods, presenting some advantages in terms of non-ionizing radiation, low implementation costs, and patient comfort [3]. Microwave imaging is carried out in terms of the contrast in the constitutive electromagnetic parameters, i.e., permittivity and conductivity, between the healthy breast tissue and cancerous tumors. This contrast implies that the presence of a tumor in the breast will cause an incident electromagnetic field to scatter, allowing the location and the reconstruction of the dielectric properties and geometries of the breast and tumors to be obtained [4,5]. Various different approaches can be applied within the framework of inverse scattering procedures by addressing data inversion in various ways that are strictly related to the specific target and/or to the image setup and the operating conditions [6].

The inverse scattering approach is complex because it is an ill-posed and non-linear problem, leading to different numerical solutions [7]. Iterative approaches are generally used to solve a nonlinear problem, regardless of whether one attempts to directly solve the original nonlinear equation or to solve an optimization problem within which the original problem is thrown. As compared to qualitative methods, quantitative approaches are usually characterized by a fairly high computational cost, but they have the advantage of providing the most complete information [8].

There have been many advances in the literature over the years, proposing various methods, such as the Rytov and the Born approximations [9,10], which are used in the case of weak scatterers. Considering some conditions, the inverse scattering problem (ISP) can be solved through various non-iterative methods. In this case, the inverse problem can be decomposed into several linear equations, and each linear equation can be solved without iteration, as in the case of the extended Born approximation method [11] and the backpropagation technique [12]. These approaches allow the reconstruction to be carried out in a very short time but result in low accuracy, especially in the presence of strong scatterers [8].

Iterative procedures have been also proposed, such as the Born iterative method (BIM) [13], the distorted Born approximation [14], which is equivalent to the Newton iteration method [15], and the modified gradient method [16]. This latter has allowed new investigations to be carried out and, together with the source-type integral equation method [17], has provided the basis for developing the contrast source inversion method (CSI) [18]. The above iterative methods, which minimize the objective function that quantifies the mismatch between the calculated and the measured data, are able to reconstruct the properties of the unknown scatterers, but they have one drawback related to the high computational cost required for reconstruction.

Researchers have proposed numerous modifications of the previous algorithms by presenting several extensions and variations, such as in [19,20,21,22,23], with the purpose of improving their performance in terms of accuracy, decreased signal noise, and reduced reconstruction time. In particular, a quadratic approach based on the BIM was developed in [24], with applications to breast image reconstruction by dividing the nonlinear problem into two linear subproblems and proposing a formulation of the inverse subproblems in terms of quadratic programming. Even if it demonstrates its effectiveness in terms of accuracy, the reconstruction time continues to be very high.

In order to overcome the above-mentioned problems, this paper proposes the adoption of convolutional neural networks (CNN) for a reconstruction scheme based on the BIM, using a quadratic programming approach. In recent years, research has emerged [25,26,27] in which the link between conventional iterative and non-iterative algorithms and deep learning networks is studied, which has allowed researchers to achieve great performance within the fields of image classification [28,29] and segmentation [30], showing good results in inverse problems when performing exact reconstructions by eliminating noise, and giving very low error rates, thus revealing that the method is very useful for biomedical applications in terms of diagnosis and therapy.

The paper is organized as follows: in Section 2, the standard inverse-scattering problem formulation, in terms of integral equations, is briefly outlined; the proposed method is presented in Section 3 by preliminarily recalling the fundamentals of the quadratic programming approach and then outlining the machine learning procedure, in terms of the CNN. Numerical validations on a circular model, as well as on breast phantoms, are discussed in Section 4. Finally, the conclusions are outlined in Section 5.

## 2. Problem Formulation

**Figure 1:**
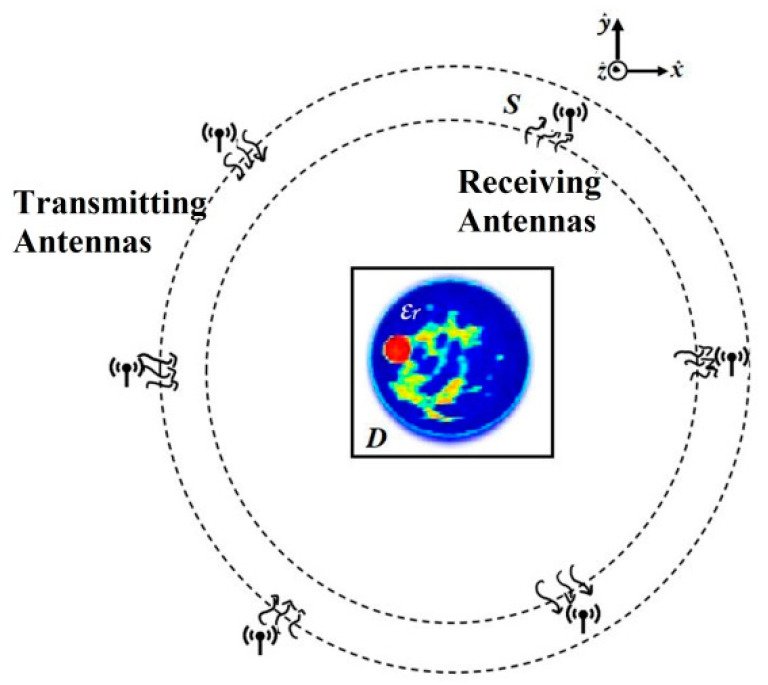
Acquisition scheme of the inverse scattering problem for breast cancer.

Referring to Figure 1, a circle is considered for the observation domain when obtaining microwave images. The circle includes the cross-section of the object to be inspected, which is sequentially illuminated through the application of a set of incident fields. These are generated either by several transmitting antennas or by a single source moving around the target [6], assuming a homogeneous background medium with permittivity ε_b_ and permeability 𝜇_0_. Non-magnetic scatterers, characterized by the relative dielectric constant ε_r_(r), are located within the domain of interest D ϵ R^2^, and illuminated by the Ni line sources, located at the point $r_{p}^{i}$ with p = 1, 2, …, Ni. For each incidence, the scattered field is measured by Nr antennas, located at the point $r_{q}^{s}$ with q = 1, 2, …, Nr.

The direct problem is described using two well-known equations. The first one is defined in terms of the electric-field integral equation, which relates the total, incident, and scattered fields (a time-dependency in the form of e^jωt^ is assumed), describing the interaction of the wave scatterer in the D-domain. It is also known as the state equation [8], and is reported as follows:

(1) $$E^{t}\left(r\right)=E^{i}\left(r\right)+k_{b}^{2}\int_{D}^{}g\left(r,r^{\prime }\right)\;J\left(r^{\prime }\right)dr^{´},\;\mathrm{for}\;r\;\in \;D\;$$

where:

− $E^{t}\left(r\right)$ is the total electric field;
− $E^{i}\left(r\right)$ is the incident electric field;
− $k_{b}=w\sqrt{\varepsilon_{b}\mu_{0\;}}$ is the wavenumber of the homogeneous medium background;
− J(r) is the contrast current density, defined as $J\left(r\right)=\chi \left(r\right)E^{t}\left(r\right)$, where the contrast function is given by:

(2) $$\chi \left(r\right)=\frac{\varepsilon_{r}\left(r\right)-1}{\varepsilon_{r_{b}}}-j\frac{\left(\sigma \left(r\right)-\sigma_{b}\right)}{\omega \varepsilon_{b}}.$$

Here, $\varepsilon_{r}$ is the relative permittivity, σ is the conductivity [S/m], ω is the angular frequency [rad/s], and the lower index ‘b’ is used to refer to the background.

In the following equation, $g\left(r,r^{\prime }\right)$ is the 2-dimensional free-space Green´s function, which is given in terms of a Hankel function of the second kind [6], namely:

(3) $$G_{2D}\left(r,r^{\prime }\right)=-\frac{j}{4}H_{0}^{\left(2\right)}\left(k_{b}\left|r-r^{\prime }\right|\right)$$

The second equation describes the scattered field in terms of the re-radiation of the induced contrast current, and is known as the data equation, namely:

(4) $$E^{s}\left(r\right)=k_{b}^{2}\int_{D}^{}g\left(r,r^{\prime }\right)\;J\left(r^{\prime }\right)dr^{\prime },\;\mathrm{for}\;r\;\in \;S$$

where $E^{s}\left(r\right)$ is the scattered field on the measurement surface *S*.

The discretized forms of Equations (1) and (4) are obtained by dividing the domain D into a square grid of M × M pixels, introducing the operators *GS*(⋅) for *r* ∈ *S* and *GD*(⋅) for *r* ∈ *D.* By following the above procedure, the following discretized equations are obtained for Equations (1) and (4), respectively:

(5) $${\bar{E}}^{t}={\bar{E}}^{i}+{\overset{=}{G}}_{D}*\bar{J}$$

(6) $${\bar{E}}^{s}={\overset{=}{G}}_{s}*\;\bar{J}$$

where the vectors ${\;\bar{E}}^{t}$, ${\;\bar{E}}^{i},{\;\bar{E}}^{s}$ represent the discretized forms of the total electrical field, the incident field, and the scattered field, respectively. The matrices $\overset{=}{G_{D}}$ and $\overset{=}{G_{s}}$ denote the free-space Green’s functions at domain *D* and boundary surface *S*, respectively [27]. Following the same procedure, the contrast χ(*r*) of domain *D* is discretized into an M^2^-dimensional form and, from the definition of the contrast current density *J*(*r*), the discretized forms of *J*(*r*) can be computed as $\bar{J}=\mathrm{diag}\left(\;\bar{\chi }\right)\cdot {\;\bar{E}}^{t}\;$ [12].

When solving the above equations, the known quantities are the incident fields ${\bar{E}}^{i}$, the matrices, $\overset{=}{G_{D}}$ and $\overset{=}{G_{s}}$, and the values of the background parameters, $\varepsilon_{b}$ and $\sigma_{b}$. The main goal of ISP is to obtain the contrast χ(r) for *r* ∈ *D*, depending on the field ${\bar{E}}^{s}\left(r\right),$ for *r* ∈ *S* and the total electric field ${\bar{E}}^{t}$ in *S*. This represents a non-linear problem, since the electric field depends on the contrast map; it is also considered an ill-framed problem that may not have a unique solution.

## 3. Method

### 3.1. Quadratic Programming Approach

In this subsection, a brief summary of the quadratic programming approach [24] to solve the BIM method [13] is presented.

The BIM method consists of a linearized iterative procedure, based on an initial assumption of the contrast map. It sequentially estimates the total electric field and the contrast function; in each iteration, the integral equations involved in the direct problem, as well as the integral equation for the contrast function related to the inverse problem, are solved [24].

The discrete model for the scattered electric field (i.e., the discretized form of Equation (4)) can be expressed as follows [6]:

(7) $$E_{m}^{s}=\overset{N}{\underset{n=1}{\sum }}g_{mn}\;\chi_{n\;\;}E_{n}^{t},\;\;m=1,\ldots ,M$$

where:

(8) $$g_{mn}=-\frac{j}{2}\pi k_{b}a_{n}\;J_{1}\left(k_{b}a_{n}\right)H_{0}^{\left(2\right)}\left(k_{b}\left|r_{m}-r_{n}\right|\right)$$

and:

- $E_{m}^{s}$ is the scattered electric field at the position *r_m_* on the surface *S*;
- *g_mn_* is the discretization of the Green function, $a_{n}=\sqrt{\Delta x\Delta y/\pi ,\;}\;J_{1}$ is the Bessel function of the first type, and *r_n_* is the vector position of the *n*-th pixel;
- $\chi_{n}$ is the contrast value at *r_n_*;
- $E_{n}^{t}$ is the total electric field at *r_n_*.

In order to determine the contrast function, denoted by $\tilde{E_{m}^{s}}$, the measured scattered field, the following minimization problem can be set up thus:

(9) $$\underset{\chi }{\min}\overset{M}{\underset{m=1}{\sum }}{\left|\tilde{E_{m}^{s}}-\overset{N}{\underset{n=1}{\sum }}g_{mn}\;\chi_{n}\;E_{n}^{t}\right|}^{2}\;\;\chi_{n}\in \mathbb{C},\;\;n=1,\ldots ,N.\;$$

This can be reformulated, in terms of a quadratic programming (QP) problem, as [24]:

(10) $$\underset{\chi }{\min}\overset{M}{\underset{m=1}{\sum }}{\left|d_{m}\right|}^{2}$$

(11) $$s.t:\;d_{m}+\overset{N}{\underset{n=1}{\sum }}g_{mn}\;\chi_{n}\;E_{n}^{t}=\tilde{E_{m}^{s}},\;\;m=1,\ldots ,M\;\chi_{n}\in \mathbb{C},\;n=1,\ldots ,N.\;d_{m}\in \mathbb{C},\;m=1,\ldots ,M.$$

This presents one global minimum, for which several efficient resolution methods exist. However, since all procedures for solving QP problems require real quantities, while complex terms are present in the problems given in Equations (10) and (11), it is necessary to perform a rearrangement in order to obtain an equivalent optimization problem, one in which only real variables and constraints are present. The rearranged problem is based on the introduction of real auxiliary variables, representing the real and the imaginary parts of the complex terms involved in Equations (10) and (11) [24]. Furthermore, in order to limit the ill-framed nature of the problem and to get more information, the $L\in {\mathbb{R}}^{+}$ incident electric fields (i.e., several sources located at different positions), defined at $F\in {\mathbb{R}}^{+}$ frequency values, are considered. Accordingly, a further rearrangement of the problem is performed, in order to take into account both the frequency dependence of the electric field and the Green function, as well as to consider multiple incident fields [24].

Although the introduction of more frequencies and more sources tends to limit the ill-framed nature of the problem, this may not be sufficient in principle to achieve reliable solutions. For this reason, it is advantageous to apply regularization procedures. In particular, the Tikhonov regularization method [31,32,33,34,35] is exploited, in which the regularization term consists of the integral of the gradient norm of the contrast function [24].

Finally, a further aspect is related to the choice of the initial solution (initial guess) for the contrast function. In an earlier work [24], two types of initial solutions are considered. The first one is the Born approximation of first-order [8], solved by setting the initial solution for the contrast at zero. The second one exploits the *L* incident electric fields, also taking into account simultaneously the different *F* frequency values chosen.

### 3.2. Proposed Machine Learning Method

**Figure 2:**
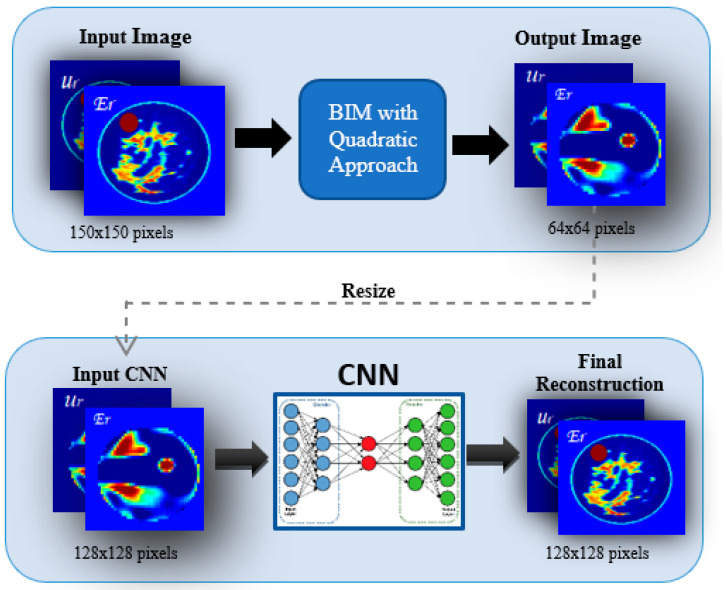
Proposed scheme with quadratic BIM and CNN.

To solve the inverse scattering problem and perform a reconstruction of the dielectric profile, a BIM with a quadratic approach is adopted, as proposed in [24,35], but instead applies a machine-learning approach based on a CNN with U-Net architecture [36]. The proposed scheme is shown in Figure 2.

The CNN used in this work, originally developed by the authors of [36], has the main objective of complementing a standard contracting network through successive layers by replacing the grouping operators with upsampling operators. This approach allows for perfect segmentation, considering large images and applying the mosaic overlay technique.

**Figure 3:**
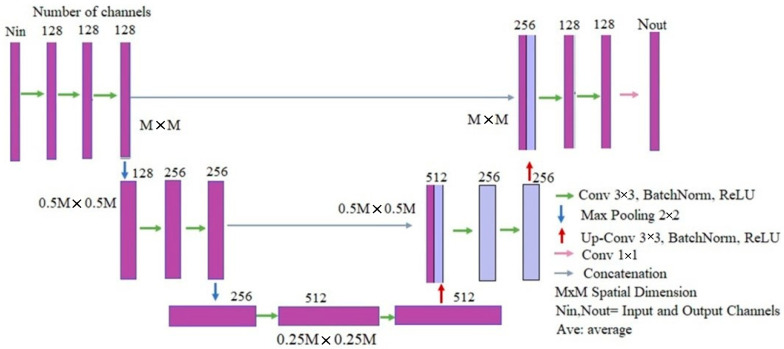
U-net CNN architecture for the proposed scheme.

The architecture of the network applied in this work is depicted in Figure 3. It is made up of a contraction part (left side) and an expansion part (right side). The left-side part is a typical convolutional network, consisting of two 3 × 3 convolutions, batch normalization, and a rectified linear unit (ReLU) for each convolution, followed by a 2 × 2 maximal pooling operation with step 2. For the purposes of downsampling, at each resolution reduction step, the number of function channels must be doubled.

The right-side part is based on upsampling, with a 2 × 2 convolution halving the number of feature map channels, a concatenation with the clipped feature map of the contraction part, and two 3 × 3 convolutions, with one ReLU for each one.

## 4. Numerical Results

### 4.1. Circular Model

**Figure 4:**
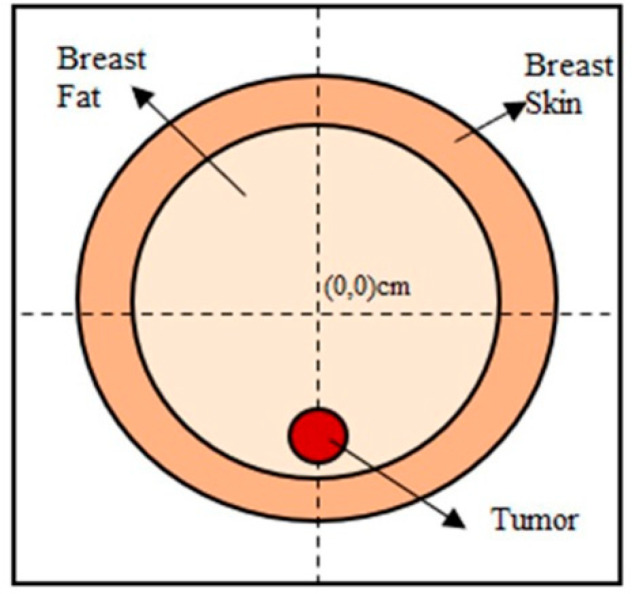
The circular breast model, used for validation.

Prior to carrying out the machine learning procedure with the breast phantoms, validation tests of the quadratic programming-based microwave imaging method were performed at a waveform frequency equal to 1 GHz, assuming a circular model for the breast. A domain of interest, D, having a size equal to 18 × 18 cm^2^, is assumed. In terms of discretization, 150 × 150 pixels were considered for the initial image, and 64 × 64 pixels were assumed for the reconstructed image. With reference to the configuration illustrated in Figure 4, 18 line sources and 18 line receivers were arranged in a circle with a radius equal to 10 cm, centered at (0.0) cm.

The model includes two large circles and one small one; this smaller circle represents a tumor, located at different positions within the larger double circle. The parameters involved in the breast model are detailed as follows:

− large circle diameter = 8 cm, medium with ε_r_ = 40;
− medium circle diameter = 6 cm, with ε_r_ = 4.5;
− small circle (tumor) diameter = 1 cm, with ε_r_ = 57.

A background medium with a relative permittivity value, ε_r_ = 10, has been assigned.

**Figure 5:**
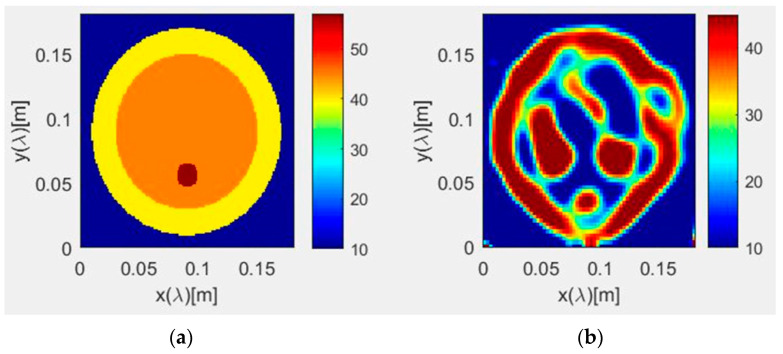
Numerical results from the quadratic programming-based microwave imaging for the circular breast model, showing the original (**a**) and retrieved (**b**) relative permittivity.

For these first validation tests, only the dielectric permittivity was retrieved. Concerning the scattered field sampling, 15 separate frequencies, ranging from 600 kHz up to 600 MHz, were considered, in 30 equispaced positions on the circular array. A set number of 10 iterations was performed for this configuration. The retrieved numerical results are reported in Figure 5, where a satisfactory agreement can be observed with the original values (Figure 5a).

### 4.2. Breast Phantoms

Full validation of the proposed machine learning approach was performed by using models of MRI-derived breast phantoms drawn from the University of Wisconsin Computational Laboratory (UWCEM) numerical breast phantom repository [37], with four different 2D models of phantoms, namely: Class 1, Phantom 1, Breast ID: 071904; Class 2, Phantom 1, Breast ID: 012204; Class 3, Phantom 2, Breast ID: 070604PA2; and Class 4, Phantom 1, Breast ID: 012304.

In the validation test, a frequency equal to 1 GHz was assumed, with a domain of interest, D, having a size equal to 18 × 18 cm^2^, namely, 3λ/5. In terms of discretization, 150 × 150 pixels were considered for the initial image, and 64 × 64 pixels were considered for the reconstructed image. In total, 18 line sources and 18 line receivers were arranged in a circle with a radius equal to 10 cm, centered at (0.0) cm. Scattered field sampling was carried out using 15 different frequencies, assuming a background relative permittivity ε_r_ = 10 [37,38,39]. A relative permittivity range of from 2.5 to 67 is assumed for the breast, with the initial solution of the contrast map using the minimum value. An image of 150 × 150 pixels was considered, from which the scattered fields were obtained. In the reconstruction, an image of 64 × 64 pixels was obtained, this being helpful for significantly reducing the execution time of the quadratic algorithm to approximately 20 min for 5 iterations. Simulations were performed for the four classes of phantoms mentioned above, by also adding three false tumors (of 3 mm, 5 mm, and 8 mm in diameter) at different locations. Specifically, the tumor was placed within the breast model, with coordinates (X cm, Y cm) in nine different positions, namely: (0 cm, 0 cm), (0 cm, 1.9 cm), (0 cm, −1.9 cm), (1.9 cm, 0 cm), (−1.9 cm, 0 cm), (1.9 cm, 1,9 cm), (−1.9 cm, 1,9 cm), (1.9 cm, −1,9 cm), and (−1.9 cm, −1,9 cm), thus obtaining a total of 108 images. These are used as input for the CNN to be trained.

To perform a quantitative evaluation of the proposed approach, the following expression for the relative error [12] is adopted:

(12) $$R_{e}=\frac{1}{N_{t}}\overset{N_{t}}{\underset{j=1}{\sum }}\|\overset{=}{\varepsilon_{r}^{o}}-\overset{=}{\varepsilon_{r}^{m}}{\|}_{F}/\|\overset{=}{\varepsilon_{r}^{o}}{\|}_{F}$$

where $\overset{=}{\varepsilon_{r}^{o}}$ is the original relative permittivity, $\overset{=}{\varepsilon_{r}^{m}}$ represents the measured relative permittivity, $N_{t}$ is the number of performed tests, and the operator ${\|}_{.}{\|}_{F}$ denotes the Frobenius norm.

Following the approach outlined in [24], a linear regression model was applied to recover the conductivity σ from the relative permittivity ε_r_ retrieved by the adopted machine learning approach, so as to finally have a full characterization of the breast phantoms. The conductivity model is expressed by the following equation [24]:

(13) $$\sigma \left(\varepsilon_{r}\right)=0.019\varepsilon_{r}-0.047.$$

**Figure 6:**
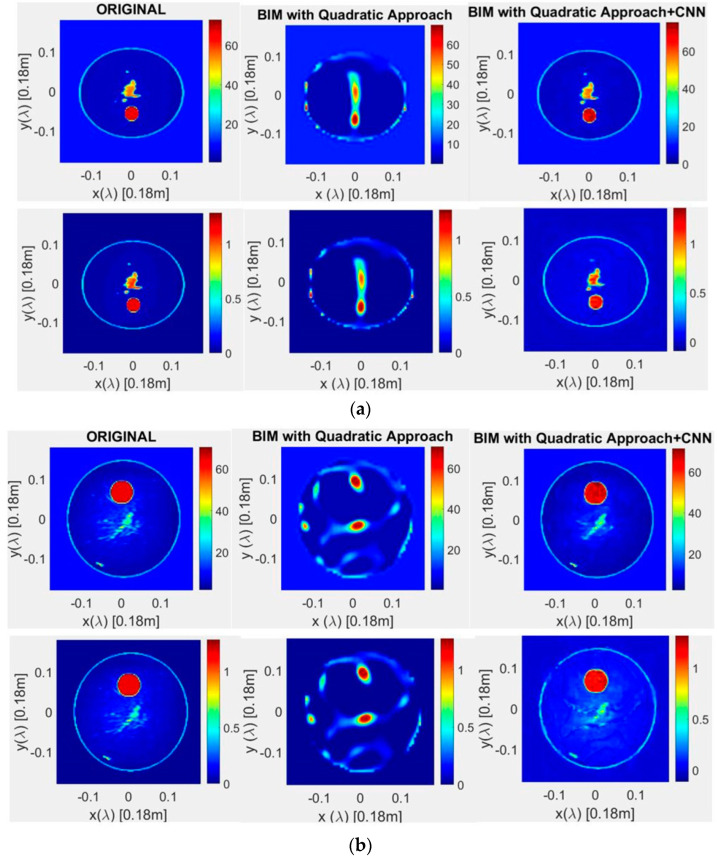

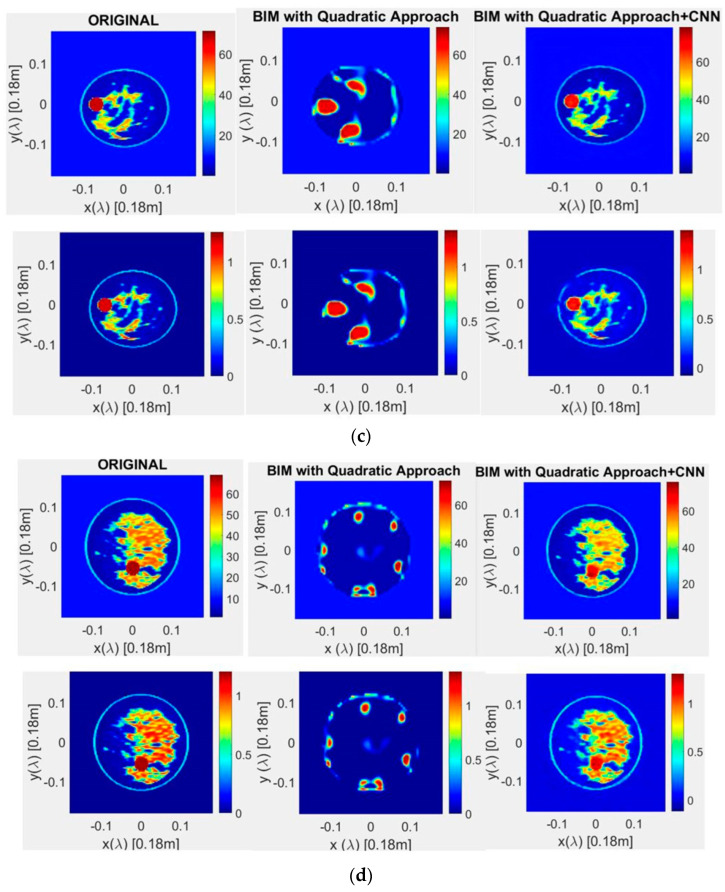
Permittivity (upper) and conductivity (lower) results, obtained with the CNN method applied to quadratic programming-based microwave imaging: (**a**) Class 1, Phantom 1, Breast ID: 071904; (**b**) Class 2, Phantom 1, Breast ID: 012204; (**c**) Class 3, Phantom 2, Breast ID: 070604PA2; (**d**) Class 4, Phantom 1, Breast ID: 012304.

In Figure 6, the reconstructed parameters for the four validation phantoms are reported and compared with the original (expected values). For all cases, the best reconstruction, which is in perfect agreement with the actual one, was achieved by applying the CNN to the BIM with a quadratic programming approach. In Table 1, a detailed comparison in terms of quantification error is reported. In particular, for all considered phantoms, a successful error decrease (below 10%) can be observed when passing from a standard quadratic BIM to the proposed approach (BIM + CNN). Furthermore, an accuracy greater than 90% was achieved with the adoption of the machine learning procedure.

## 5. Conclusions

A machine learning approach based on CNNs has been proposed in this work to solve inverse scattering problems, formulated in terms of a quadratic programming-based BIM. Compared to most available algorithms, which work easily on weak scatterers, the introduced method can be applied to strong scatterers, which are very common in medical applications. Validation tests have revealed that the proposed machine learning technique is able to reconstruct the relative permittivity and the conductivity from calibrated experimental data within a time of approximately 15 min, with an accuracy greater than 90%. In future studies, the proposed method will be applied for the relative permittivity reconstruction of other tomographic images in the medical field, such as those relating to brain tumor phantoms. Furthermore, to obtain full experimental validation, a test setup will be implemented in the ERMIAS Laboratory at the University of Calabria, by realizing laboratory gel-like phantoms [40] and performing scattering measurements.

## Figures and Tables

**Table 1:** Quantification error of the validation tests for the circular model and breast model.

	Error Quadratic BIM	Error Quadratic BIM + CNN	Accuracy Quadratic BIM + CNN
Circular model	44%	-	
Class 1, Phantom 1	47.1%	7.6%	92.4%
Class 2, Phantom 1	74.9%	9.8%	90.2%
Class 3, Phantom 2	78.8%	7,9%	92.1%
Class 4, Phantom 1	88.5%	7.05%	92.95%

## Data Availability

The data presented in this study are available on request from the corresponding author.
